# The first series of cases of ketosis-prone type 2 diabetes (flatbush diabetes) in Brazilian adults

**DOI:** 10.20945/2359-3997000000329

**Published:** 2021-02-25

**Authors:** Luana Aparecida de Lima Ramaldes, Sarah Simaan dos Santos, João Roberto de Sa, Patrícia Médici Dualib, Sérgio Atala Dib

**Affiliations:** 1 Universidade Federal de São Paulo Departamento de Medicina Centro de Diabetes São Paulo SP Brasil Departamento de Medicina, Disciplina de Endocrinologia, Centro de Diabetes, Universidade Federal de São Paulo, São Paulo, SP, Brasil

## Abstract

Ketosis-prone type 2 diabetes (KPD) is an emerging form of diabetes mellitus characterized by unprovoked ketoacidosis, absence of autoimmunity and beta-cell dysfunction. The KPD may improve after initial glycemic compensation and evolve to exogenous insulin independence, most cases were observed in populations with African or Hispanic backgrounds. We reviewed the literature on KPD and, to date, only one case of KPD has been described in Brazil's multi-ethnic population. A group of adult Brazilian KPD patients without autoimmunity and insulinopenia was identified for this study. We report a retrospective study of four KPD cases (3 males) evaluated in southeast Brazil, the patients were overweight or obese, age between the third and fifth decades of life, had a family history of type 2 diabetes, hyperglycemia (809.5 ± 344.2 mg/dL), acidosis (pH 7.21 ± 0.07; normal range (nr): 7.35-7.45 and bicarbonate 9.1 ± 6.2; nr: 22-26 mEq/mL), ketonuria (142.5 ± 114.4 mg/dL; nr: absence), absence of glutamic acid decarboxylase antibodies (GAD-65), and beta-cell function reserve (C-peptide 1.19 ± 0.53 ng/mL - nr: 1.1-4.4 ng/mL) on diagnosis. After glycemic compensation, there was increase of C-peptide (2.21 ± 0.41) indicating the recovery of beta-cell function and the time to insulin independence was 7.7 ± 3.5 months. They evolved after the period of glucotoxicity with insulin withdrawal and could be treated with oral antidiabetic therapy. This is the first case series of KPD described in Brazil being characterized by ketoacidosis at diagnosis, absence of autoimmunity, recovery of beta-cell function and insulin independence.

## INTRODUCTION

Diabetes mellitus (DM) comprises a group of metabolic diseases characterized by hyperglycemia resulting from a defect in insulin secretion, insulin action, or both. Diabetes Mellitus that emerges with ketoacidosis in adults may correspond to a heterogeneous group of diseases with different etiologies (1–5). One of these pathologies, ketosis-prone type 2 diabetes (KPD), is associated with severe hyperglycemia and ketoacidosis on presentation, followed by remission after insulin therapy (5,6). After weeks or months, these patients can discontinue insulin and maintain glycemic control using diet or oral agents or both. These patients do not have anti-islet autoantibodies, but may have some common type 1 diabetes susceptibility *HLA* alleles (7,8).

Ketosis-prone diabetes mellitus was initially described in the 1960s in the United States (9), followed by reports in Africa, Japan, Spain, China, and Pakistan, suggesting a global distribution of the disease (2). The prevalence of KPD cases is higher in African Americans and Hispanics than in Caucasians and Asians (10). In addition, is also an important differential diagnosis among individuals classified as having adult type 1 diabetes, as it affects individuals over 30 years old, often with the first decompensation in ketoacidosis.

In Brazil, the population has a multi-ethnic origin, with over 70% of the population composed of Europeans and Africans descendants (11). Nevertheless, despite the population's African background and the importance of patients and clinicians’ recognition and understanding of this type of diabetes, the Brazilian national literature contains only one case reported of KPD (12). We report a retrospective study of a series of cases and a literature review of KPD.

## DESCRIPTION OF CASES

### General characteristics

The patients were recruited from March to November 2018 by retrospective data analysis of the last 6 years, all patients were followed in the Diabetes Center of the Federal University of São Paulo, Brazil. The literature review and data reassessment were carried out in March 2020. The KPD diagnosis was based on the occurrence of spontaneous severe ketoacidosis in individuals with diabetes diagnosis not classifiable in the classic diabetes types, age above 30 years, absence of antiglutamic acid decarboxylase antibodies (GAD-65) and beta-cell function reserve after correction of ketoacidosis (fasting C-peptide level least 1 ng/mL; normal range [nr]: 1.1-4.4 ng/mL; immunofluorometric assay (Auto Delfia, Finland) with a detection limit of 0.10 ng/mL), criteria previously established by Maldonado and cols. (2). The diagnosis of ketoacidosis was defined as a blood pH ≤ 7.3 within the first 24h, with positive urine ketones. The institution's ethics committee approved the study (CAAE NUMBER: 96720818.6.0000.5505), and informed consent term were obtained from the participants.

The general clinical and biochemical characteristics of the studied patients appear in Table 1, and follow-up data in Table 2.

**Table 1:** Clinical and biochemical characteristics at diagnosis of ketosis prone diabetes in Brazilian patients

Clinical Parameters	Case 1	Case 2	Case 3	Case 4	TOTAL
Age at diagnosis (year)	45	48	42	30	41 ± 7.6
Gender	Male	Male	Male	Female	75% (Male)
Ethnicity	Pardo	White	Pardo	Black	
Weight loss > 5%	Yes	Yes	Yes	Yes	100%
Family History of Type 2 Diabetes	Yes	Yes	Yes	Yes	100%
BMI (kg/m²)	39.9	25.5	28.4	27.7	30.4 ± 6.5
Acanthosis nigricans	Yes	No	Yes	Yes	75%
Arterial Hypertension	Yes	Yes	Yes	No	75%
**Laboratory Parameters**					
HbA1C (%)	14.3	17.1	15.1	10.5	14.2 ± 2.8
Serum Glucose (mg/dL)	922	413	1221	682	809.5 ± 344.2
pH	7.28	7.15	7.25	7.15	7.21 ± 0.07
Bicarbonate (mmol/L)	14.6	2.9	14.4	4.7	9.1 ± 6.2
Ketonuria (mg/dL)	80	150	40	300	142.5 ± 114.4
Total cholesterol (mg/dL)	354	126	334	133	236.7 ± 124.1
Triglycerides (mg/dL)	1129	126	742	163	540 ± 483.5
LDL-c (mg/dL)	NA*	74	NA*	46	60 ± 20
HDL-c (mg/dL)	27	27	33	54	35.2 ± 2.8
GAD-65†	NEG‡	NEG‡	NEG‡	NEG‡	
Fasting Serum C-peptide (ng/mL)	0.70	1.03	1.09	1.96	1,19 ± 0.53

NA* not available

† anti-glutamic acid decarboxylase autoantibody

‡ NEG: negative.

**Table 2:** Current treatment of ketosis prone diabetes in Brazilian patients

Current Treatment: diet and physical activity plus	Case 1	Case 2	Case 3	Case 4	Total
Time from DM* diagnose (year)	2	3	3	6	3.5 ± 1.7
Time to insulin independence (months)	4	6	9	12	7.7 ± 3.5
Metformin (g)	1.5	0.5	1.7	0.8	1.1 ± 0.5
Gliclazide (mg)	–	30	–	60	–
Dose Insulin (UI/kg)	–	–	0.5	–	–
Atorvastatin (mg)	40	No statin	10	No statin	–
Current HbA1C (%)	6.4	5.1	8.2	6.8	6.62 ± 1.1
Current Fasting Serum C-peptide (ng/mL)	2.59	1.38	2.45	2.44	2.21 ± 0.41
ΔFasting Serum C-peptide basal† (%)	370	25	224	124	221 ± 41
Retinopathy	No	Yes	No	No	25%
Microalbuminuria	Yes	Yes	No	No	50%

DM* Diabetes mellitus;

Δ Fasting Serum C-peptide basal

† (current serum C-peptide/serum C-peptide on diagnosis) X100.

## CASE 1

A 48-year-old male who was diagnosed with diabetes mellitus at age 45 presented in our emergency room with symptoms of glycemic decompensation (polydipsia, polyuria, and weight loss). His laboratory tests revealed hyperglycemia (922 mg/dL), acidosis (pH 7.28; nr: 7.35-7.45) and ketonuria positive without an identified triggering factor. His glycosylated hemoglobin (HbA1c) was 14.3%, and he had hypertriglyceridemia (TG:1129 mg/dL). Pancreatitis was excluded based on the clinical picture and normal values of lipase and amylase in his blood. Regarding the etiological diabetes investigation, GAD-65 autoantibody was negative, and the patient's fasting C-peptide level was 0.70 ng/mL. During his first outpatient clinic visit, he denied previous comorbidities but indicated a family history of two generations affected by DM. Physical examination revealed a BMI of 39.9 kg/m^2^ and severe cervical acanthosis. After 8 weeks of insulin treatment, he began to present episodes of hypoglycemia and required a reduction of the initial insulin dose. After 12 weeks of follow-up, his laboratory tests revealed: HbA1c 6.1%, fasting C-peptide level 1.21 ng/mL. Due to the absence of GAD-65 autoantibodies and fasting C-peptide elevation after improved glycemic control, there was suspicion of KPD, thus his treatment was modified for metformin and lifestyle changes. Tables 1 and 2 summarize these data.

## CASE 2

A 51-year-old male was monitored in the Diabetes Center's outpatient clinic since the diagnosis of diabetic ketoacidosis (Table 1) at age 48 [HbA1c 17.1%, GAD-65 autoantibody negative, and fasting C-peptide level 1.03 ng/mL], without triggering factor identified. Regarding antecedents, he indicated an absence of comorbidities except social alcohol consumption and a family history of DM. On physical examination, he presented a BMI of 25.5 kg/m^2^ without other significant abnormalities. After 9 weeks of insulin treatment, he began to have episodes of hypoglycemia, so we began a progressive reduction of his insulin dose. After 16 weeks of treatment, his HbA1c was 6.4%, and fasting C-peptide level was 1.38 ng/mL. Six months later, the insulin therapy was stopped, and he started 500 mg of metformin and 30 mg of gliclazide per day. So far, he is doing well during follow-up.

## CASE 3

A 45-year-old male in follow-up at the Diabetes Center's outpatient clinic referred to the diagnosis of DM at age 42 when presenting symptoms of glycemic decompensation (polydipsia, polyuria, and weight loss) and unprovoked ketoacidosis. At diagnosis, his laboratory exams revealed blood glucose 1221 mg/dL, HbA1c 15.1%, high lipids (total cholesterol – 334 mg/dL); triglycerides: 742 mg/dL), fasting C-peptide level 1.09 ng/mL, and GAD-65 autoantibodies negative. He referred to a previous diagnosis of systemic arterial hypertension and a family history of DM. Physical examination showed a BMI of 28.4 kg/m^2^ and mild cervical *acanthosis nigricans*. After 9 weeks of treatment, the doses of insulin necessary to establish glycemic control began to decrease, and after 18 weeks, his HbA1c had decreased to 7.8% and his fasting C-peptide level had increased to 4.5ng/mL. At his last appointment, he was on basal insulin (0.5U/kg/day) plus metformin (1.7 g/day) and atorvastatin (10 mg/day).

## CASE 4

A 36-year-old female had been undergoing follow-up at the Diabetes Center outpatient clinic since her DM diagnosis. She was diagnosed at age 30, presenting symptoms of glycemic decompensation (polydipsia, polyuria, and weight loss), acidosis, and ketonuria, without an identified triggering factor (Table 1). At diagnosis, her exams test revealed: HbA1c 10.5%, negative for GAD-65 autoantibodies and fasting C-peptide 1.96 ng/mL. During her first outpatient clinic visit, she indicated no history of previous comorbidities, but mentioned a family member with a recent DM diagnosis. On physical examination, she presented a BMI of 30.3 kg/m^2^ and moderate cervical *acanthosis nigricans.* After 8 weeks of insulin treatment, she began to present periods of hypoglycemia, so her insulin daily dose was decreased. After 12 months, insulin was discontinued. By this time, her exam tests revealed: HbA1c 6.2%, fasting C-peptide 1.9 ng/mL and post-prandial C-peptide level was 7.2 ng/mL, thus her treatment was modified for 850 mg of metformin and 60 mg of gliclazide per day. This patient maintained oral antidiabetic treatment for 2 years, after that time the medications were withdrawn, and she got good glycemic control only with diet and exercise. However, in last year, she presented some episodes suggestive of minor ketosis hyperglycemia decompensation, making it necessary guidelines for its correction with insulin and prescription of oral medication. A resume of the four patients’ current treatment is shown in Table 2.

In summary, the initial treatment of all patients included insulin, which was discontinued after periods varying from 4 to 12 months, with exception of one patient who required a low dose of insulin for maintenance after initial compensation.

## DISCUSSION

Ketosis-prone diabetes is being recognized worldwide. A review of the theme demonstrates the prevalence according to ethnicity, with a high risk of occurrence in the African American and Hispanic populations and low risk in Caucasian and Asian populations (10), but data are insufficient to define the exact prevalence. In Brazil, a recent study showed that the Brazilian population has a predominance of European and African ancestry (11), but only one case report (12) of KPD in more than a decade, so this type of diabetes may be underdiagnosed in Brazil.

Here we present a series of cases of KPD cases from southeast Brazil. The diagnosis was made in overweight or obese males between the third and fifth decades of life with characteristics comparable to African, Hispanic, Caucasian, and Asian American individuals.

These patients’ most important phenotypic characteristics include spontaneous ketoacidosis, but the mechanisms responsible for this unprovoked ketoacidosis are not completely known. At least five important abnormalities have been discussed in this respect: A) pancreatic beta-cell function is very sensitive to glucotoxicity (13) because of the low expression of antioxidant enzymes necessary for the elimination of reactive oxygen species; B) there is a higher prevalence of G6PD deficiency (related to anti-oxidant defense) in KPD compared with controls and patients with classical type 2 diabetes (14); C) alpha-cell dysfunction is similar to classical type 2 diabetes, resulting in inappropriate secretion of glucagon (15); D) ketolysis (clearance of ketone bodies) decreases (16); and E) prolonged ketonemia *per se* could exert negative effects on beta-cell function in patients with KPD (17).

The insulin resistance component in patients with KPD has also been discussed; some studies show that it can be important when evaluated shortly after the initial event but increases slightly or is persistent when associated with obesity after hyperglycemia resolution during follow-up (14). All our patients had a family history of type 2 diabetes and presented clinical characteristics associated with insulin resistance, such as obesity and the presence of *acanthosis nigricans*. In addition, 3 of 4 had glycemia (> 500 mg/dL) and 2 of 4 had hypertriglyceridemia (> 500 mg/dL) at the time of diagnosis. This last found in our patients, in favor of insulin resistance was not highlighted in previous studies. This might collaborate to β dysfunction because some researchers suggest that a period of exposure to high levels of glucose and fatty acids can result in significant impairment of insulin secretion in individuals with a family history of type 2 diabetes (18,19). However, recent studies have shown that a decrease in beta-cell function during the acute phase of the disease is related more to the toxic effects of chronically elevated glucose levels (glucotoxicity) than to high lipid concentrations; in other words, pancreatic islet patients with KPD might have a resistance to lipotoxicity (18).

With the improvement of hyperglycemia, we can detect the recovery of beta-cell function in our patients (3 of 4 patients had an increase of more than 100% in basal C-peptide levels on follow-up). Case 2 was the only patient who had a normal weight, lacked *acanthosis nigricans*, and had a high HbA1c level, at diagnosis, and experienced a smaller increase in insulin secretion (only 25% of basal) and normal lipid profile at follow-up (in opposition to insulin resistance).

Significant recovery of beta-cell function was reported, and insulin independence in the group was higher than 75%. Only Case 3 maintained the use of insulin to ensure adequate glycemic control, which is compatible with the findings of other studies that characterized a cohort of 223 KPD patients, only 23% of whom maintained insulin dependence (3). However, the persistence of residual insulin beta-cell secretions for more than 3 years of DM diagnosis was observed in all our patients.

Given the discussion above and given that the expression of those conditions are variable in KPD, after the resolution of beta-cell glucotoxicity, treatment with oral medications commonly used in type 2 DM is possible.

In our study, the use of insulin, metformin, and sulfonylurea (gliclazide) stands out mainly due to the limitation of low-cost antidiabetic drugs available at our public hospital, but so far as possible get a satisfactory glycemic control.

Reported cases describe the use of modern therapies such as DPP4 inhibitors, SGLT2 transporter inhibitors, and GLP1 analogs (20). Nevertheless, is important to mention that if SGLT2 transporter inhibitors are used in this group of patients, we must be cautious because these patients have an increased risk of ketoacidosis (15) which is a possible complication of these drugs due to various pathophysiological mechanisms (21). These include increased glucagon secretion and the consequent rise in lipolysis and ketogenesis, as well as the reduction in renal ketone clearance.

## CONCLUSIONS

This is the first case series of KPD described in Brazilian adult with diabetes mellitus. We show that these patients classified as having atypical diabetes could also occur in the Brazilian population. KPD manifests not due to a loss of beta-cell mass, but a transitory failure of these cells to respond to hyperglycemia. Further studies are necessary to verify the prevalence of this subtype of diabetes in the Brazilian population. Therefore, when ketosis is present at diagnosis, in the absence of autoimmunity and the presence of beta-cell function, patients may be able to discontinue insulin and maintain glycemic control using appropriate diet, exercise, and oral hypoglycemic agents in follow-up.
